# Unusual Interferon Gamma Measurements with QuantiFERON-TB Gold and QuantiFERON-TB Gold In-Tube Tests

**DOI:** 10.1371/journal.pone.0020061

**Published:** 2011-06-08

**Authors:** Richard D. Powell, William C. Whitworth, John Bernardo, Patrick K. Moonan, Gerald H. Mazurek

**Affiliations:** 1 Division of Tuberculosis Elimination, Centers for Disease Control and Prevention, Atlanta, Georgia, United States of America; 2 The Pulmonary Center, Boston University School of Medicine, Boston, Massachusetts, United States of America; McGill University, Canada

## Abstract

**Introduction:**

Interferon gamma (IFN-γ) release assays, such as QuantiFERON®-TB Gold test (QFT-G) and QuantiFERON®-TB Gold In-Tube test (QFT-GIT) are designed to detect *M. tuberculosis* (*Mtb*) infection. Recognition of unusual IFN-γ measurements may help indicate inaccurate results.

**Methods:**

We examined QFT-G and QFT-GIT results from subjects who had two or more tests completed. We classified unusual IFN-γ measurements as: 1) High Nil Concentration (**HNC**) when IFN-γ concentration in plasma from unstimulated blood exceeded 0.7 IU/mL; 2) Low Mitogen Response (**LMR**) when Mitogen Response was <0.5 IU/mL; 3) Very Low Mitogen Response (**VLMR**) when Mitogen Response was ≤−0.5 IU/mL; and 4) Very Low Antigen Response (**VLAR**) when the response to a Mtb antigen was ≤−0.35 IU/mL and ≤−0.5 times the IFN-γ concentration in plasma from unstimulated blood.

**Results:**

Among 5,309 results from 1,728 subjects, **HNC** occurred in 234 (4.4%) tests for 162 subjects, **LMR** in 108 (2.0%) tests for 85 subjects, **VLMR** in 22 (0.4%) tests for 21 subjects, and **VLAR** in 41 (0.8%) tests for 39 subjects. QFT-GIT had fewer **HNC**, **VLMR**, and **VLAR** (p = 0.042, 0.004, and 0.067 respectively); QFT-G had fewer **LMR** (p = 0.005). Twenty-four (51.6%) of 47 subjects with positive results and **HNC** were negative or indeterminate by all other tests. Thirteen (61.9%) of 21 subjects with positive results and **LMR** were negative or indeterminate by all other tests.

**Conclusion:**

Unusual IFN-γ measurements including **HNC**, **LMR**, **VLMR**, and **VLAR** were encountered in small numbers, and in most instances were not seen on simultaneously or subsequently performed tests. To avoid erroneous diagnosis of *Mtb* infection, IGRAs with unusual IFN-γ measurements should be repeated with another blood sample and interpreted with caution if they recur.

## Introduction

Interferon gamma (IFN-γ) release assays (IGRAs), such as the QuantiFERON-TB Gold test (QFT-G) and QuantiFERON-TB Gold In-Tube test (QFT-GIT) are being used as substitutes for the tuberculin skin test (TST) to detect *M. tuberculosis* (*Mtb*) infection with increasing frequency [1]–[3]. These whole blood IGRAs depend on measurement of IFN-γ released from sensitized lymphocytes in whole blood incubated with specific *Mtb* antigens.

For QFT-G and QFT-GIT, aliquots of heparinized fresh whole blood are incubated with *Mtb* antigens, with mitogen, and with no antigen [4], [5]. Plasma is harvested and the concentration of IFN-γ ([IFN-γ]) is determined by enzyme-linked immunosorbent assay (ELISA). The amount of IFN-γ released is determined by subtracting the [IFN-γ] in plasma from unstimulated blood ([Nil]) from the [IFN-γ] in the plasma from blood stimulated with *Mtb* antigen (“Antigen Response”), or mitogen (“Mitogen Response”). QFT-GIT measures response to a single mixture of peptides representing two whole *Mtb* proteins called early secretory antigenic target 6 (ESAT-6) and culture filtrate protein 10 (CFP10), and part of a third *Mtb* protein called TB7.7 [6]. For QFT-G, *Mtb* antigens consist of two separate mixtures of peptides that represent either ESAT-6 or CFP10 that are used to stimulate two separate aliquots of blood. Tests are considered positive when the response to a *Mtb* antigen exceeds a predefined amount (e.g. ≥0.35 IU/mL and ≥50% of [Nil]) although interpretation criteria have changed for QFT-G [4], [6], [7] and QFT-GIT [5], [8]–[10] since their introduction. Mitogen-stimulated blood serves as a positive control for both tests. A low Mitogen Response (i.e. <0.5 IU/mL) may occur with insufficient lymphocytes, incorrect addition of the Mitogen, labeling errors, reduced lymphocyte activity due to prolonged specimen transport, improper specimen handling, or the presence of antibodies to IFN-γ [11]. The blood incubated without antigen serves as a negative control. Elevated levels of IFN-γ in the negative control (i.e. >0.7 IU/mL) may occur with incorrect addition of antigens, labeling errors, the presence of heterophile antibodies, or non-specific IFN-γ secretion. Currently, these IGRAs may be interpreted as positive despite a low Mitogen Response or high [Nil] if the measured response to a *Mtb* antigen exceeds the predefined cut point.

While these tests offer potential advantages over TST, technical errors may affect their accuracy. Recognition of unusual IFN-γ measurements may help indicate inaccurate results. We developed criteria for classifying IFN-γ measurements as “unusual” based on cut point values used to interpret QFT-G or QFT-GIT.

The objectives of this study were 1) to analyze a large population of subjects who had multiple QFT-G and/or QFT-GIT tests, 2) to determine the frequency of unusual IFN-γ measurements, 3) to determine how often unusual IFN-γ measurements occurred in multiple tests for a subject, and 4) to determine which of the two test types had more unusual IFN-γ measurements.

## Methods

### Ethics Statement

The data included in this analysis were collected from multiple studies conducted by the US Centers for Disease Control and Prevention (CDC) from June 2003 to December 2008, including three published reports [7], [12], [13]. Following CDC Human Subjects Institutional Review Board approval, people being screened for employment; people with symptoms, signs, or radiographic evidence suggestive of tuberculosis; and people with suspected exposure to *Mtb* were enrolled after providing written informed consent. After providing an initial blood sample for QFT-G and QFT-GIT, subjects were asked to return 2 to 12 weeks later to provide a second blood sample for repeat tests. Results from subjects with at least two tests were included in this analysis. The data were analyzed anonymously.

### IGRAs

For QFT-G, test antigens consisted of two mixtures of overlapping peptides representing the entire ESAT-6 protein or CFP10 protein [7]. Antigens, saline (for nil control), or phytohemagglutinin A (PHA; for mitogen control) were added simultaneously to 1 mL aliquots of heparinized blood in Costar® 24-well microtiter plates. Plates were incubated within 12 hours of collection for 16 to 24 hours at 37°C and plasma was harvested.

For QFT-GIT, 1 mL of blood was collected into 3 tubes containing only heparin (nil control); heparin, dextrose, and PHA (mitogen control); or heparin, dextrose, and *Mtb* antigens. *Mtb* antigens for QFT-GIT consisted of a single mixture of peptides representing ESAT-6, CFP10, and part of TB7.7 (Rv 2654, peptide 4) in one tube [14]–[16]. Blood was incubated within 12 hours of collection for 16 to 24 hours at 37°C prior to harvesting plasma.

For QFT-G and QFT-GIT, the concentration of IFN-γ in 50 µl of each plasma sample was determined by ELISA as previously described for the QuantiFERON®-TB Gold test [7]. Plasmas for QFT-G and QFT-GIT from the same blood sample were assayed at the same time and on the same ELISA plate. The Mitogen Response was calculated by subtracting the IFN-γ concentration in plasma from unstimulated blood ([Nil]) from the IFN-γ concentration in plasma from mitogen-stimulated blood. For QFT-G, antigen responses were calculated by subtracting [Nil] from the IFN-γ concentration in plasma from blood stimulated by ESAT-6 (“ESAT-6 Response”) and CFP10 (“CFP10 Response”); the higher of the ESAT-6 Response or CFP10 Response was used as the TB Response to interpret QFT-G as described in Table 1. For QFT-GIT, the antigen response was calculated by subtracting [Nil] from the IFN-γ concentration in plasma from blood stimulated by the single cocktail of peptides representing ESAT-6, CFP10, and part of TB7.7, and this was used as the TB Response to interpret QFT-GIT as described in Table 1.

**Table 1:** Interpretation Criteria used for QuantiFERON-TB Gold and QuantiFERON-TB Gold In-Tube^a^ Tests.

Interpretation	[Nil]^b^	TB Response^c^ ^d^	Mitogen Response^e^
Positive	Any	≥0.35 IU/mL and ≥50% of [Nil]	Any
Negative	≤0.7 IU/mL	<0.35 IU/mL	≥0.5 IU/mL
Indeterminate	≤0.7 IU/mL	<0.35 IU/mL	<0.5 IU/mL
	>0.7 IU/mL	<50% of Nil^c^	Any

a Interpretation criteria used for QFT-GIT differed from that approved by the FDA in Nov, 2008.

b “[Nil]” is the IFN-γ concentration in plasma from unstimulated blood.

c “TB Response” for the QuantiFERON-TB Gold test is the higher IFN-γ concentration in plasma from blood stimulated by a cocktail of peptides representing ESAT-6 or CFP10, minus [Nil].

d “TB Response” for the QuantiFERON-TB Gold In-Tube test is the IFN-γ concentration in plasma from blood stimulated by a single cocktail of peptides representing ESAT-6, CFP10, and part of TB7.7 minus [Nil].

e “Mitogen Response” is the IFN-γ concentration in plasma from mitogen stimulated blood minus [Nil].

### Classification of Unusual IFN-γ Measurements

We classified unusual IFN-γ measurements as: 1) High Nil Concentration (**HNC**) when IFN-γ concentration in plasma from unstimulated blood exceeded 0.7 IU/mL; 2) Low Mitogen Response (**LMR**) when Mitogen Response was <0.5 IU/mL; 3) Very low Mitogen Response (**VLMR**) when Mitogen Response was ≤–0.5; and 4) Very Low Antigen Response (**VLAR**) when a response to a *Mtb* antigen was ≤–0.35 IU/mL and ≤–0.5 times the IFN-γ concentration in plasma from unstimulated blood. These categories were not mutually exclusive. The cut points were derived from cut point values used to interpret QFT-G and/or QFT-GIT. The frequencies of the unusual IFN-γ measurements were then determined.

### Statistical Analysis

Statistical analysis was conducted using SPSS (v15.0.1.1, Chicago, Ill). Differences in the proportion of unusual IFN-γ measurements observed with QFT-G and QFT-GIT were assessed using unadjusted Mantel-Haenszel odds ratios and the Pearson Chi-Square test. *P* values ≤0.05 were considered significant.

## Results

Test results were available for more than one test for 1,728 subjects. Of these, 181 were suspected to have tuberculosis, 683 were being tested after suspected exposure to tuberculosis, and 864 were being screened for employment. Results from 4 tests (initial QFT-G, repeat QFT-G, initial QFT-GIT, and repeat QFT-GIT) were available for 841 subjects; results from 3 tests were available for 171 subjects; and results from 2 tests were available for 716 subjects. Of the 5,309 test results examined, 711 (13.4%) results were positive for *Mtb* infection; 4,338 (81.7%) results were negative; and 260 (4.9%) were indeterminate. One or more tests were positive for 325 (18.8%) subjects including 106 (58.6%) subjects suspected to have TB, 198 (29.0%) subjects with suspected exposure to tuberculosis, and 21 (2.4%) subjects being screened for employment.

### Frequency of Unusual IFN-γ Measurements

**Figure 1:**
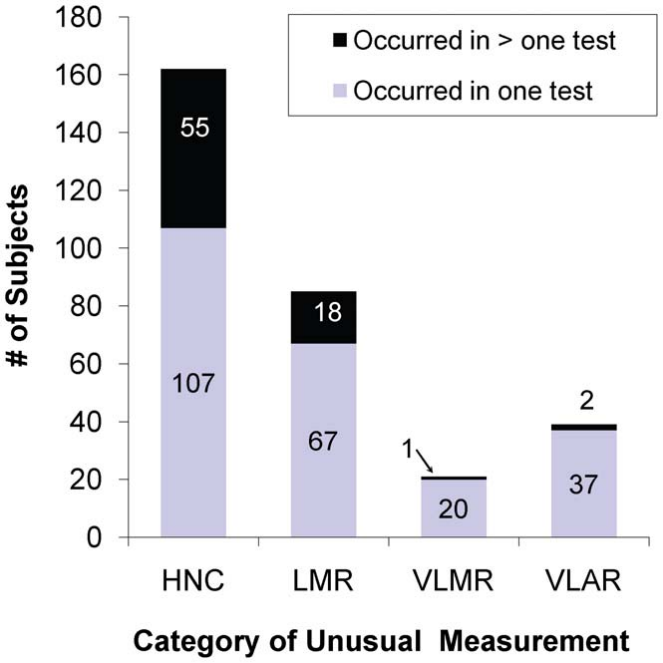
Number of subjects with unusual IFN-γ measurements. The number of subjects with unusual IFN-γ measurements categorized as High Nil Concentration (**HNC**), Low Mitogen Response (**LMR**), Very Low Mitogen Response (**VLMR**), and Very Low Antigen Response (**VLAR**) that occurred once (dark gray bars) or more than once (light gray bars) among 1,725 subjects who had 5,309 QuantiFERON-TB Gold and QuantiFERON-TB Gold In-Tube Tests performed.

Of the 5,309 results (Table 2), **HNC** occurred in 234 (4.4%) tests for 162 subjects; **LMR** occurred in 108 (2.0%) tests for 85 subjects; **VLMR** occurred in 22 (0.4%) tests for 21 subjects, and **VLAR** occurred in 41 (0.8%) tests for 39 subjects. Fifty-four tests had more than one unusual IFN-γ measurement. Fewer **HNC** and **VLMR** were observed with QFT-GIT than QFT-G (p = 0.042 and 0.005, respectively). Fewer **VLAR** were observed with QFT-GIT than QFT-G, but this difference was not significant (p = 0.067). Conversely, significantly fewer **LMR** were observed with QFT-G than QFT-GIT (p = 0.004). As illustrated in Figure 1, **HNC** occurred more than once in 55 (34.0%) of 162 subjects with **HNC**; and **LMR** occurred more than once in 18 (21.2%) of 85 subjects with **LMR; VLMR** occurred multiple times in only 1 (4.8%) of 21 subjects with **VLMR**; **VLAR** occurred multiple times in 2 (5.1%) of 39 subjects with **VLAR**. The number of subjects in each subject group with 1 or more **HNC**, **LMR**, **VLMR**, and **VLAR** differed significantly (Table 3).

**Table 2:** Frequency of Unusual IFN-γ Measurements with QuantiFERON-TB Gold and QuantiFERON-TB Gold In-Tube Testing.

	QFT-G^a^	QFT-GIT^b^	Total^c^	OR (95% CI)^d^	*p* value
# of tests	2741	2568	5309		
# of subjects	1696	1653	1728		
# (%) of tests with **HNC** e	136 (5.0)	98 (3.8)	234 (4.4)	1.32 (1.01–1.72)	0.042
# (%) of subjects with **HNC**	122	83	162		
# (%) of tests with **LMR** f	41 (1.5)	67 (2.6)	108 (2.0)	0.57 (0. 38–0.84)	0.004
# (%) of subjects with **LMR**	38	59	85		
# (%) of tests with **VLMR** g	18 (0.7)	4 (0.2)	22 (0.4)	4.24 (1.42–12.54)	0.005
# (%) of subjects with **VLMR**	17	4	21		
# (%) of tests with **VLAR** h	27 (1.0)	14 (0.5)	41 (0.8)	1.82 (0.95–3.47)	0.067
# (%) of subjects with **VLAR**	27	14	39		
# (%) of tests with any 1 or more Unusual IFN-γ Measurements	171 (6.2)	164 (6.4)	335 (6.3)		
# (%) of subjects with any 1 or more Unusual IFN-γ Measurements	154	140	236		

a “QFT-G” is QuantiFERON-TB Gold test.

b “QFT-GIT” is QuantiFERON-TB Gold In-Tube test.

c “Total # of subjects” with unusual measurement may be less than the sum of the “# of subjects” with unusual QFT-G and QFT-GIT measurements because subjects may have unusual measurements with both tests.

d “OR (95% CI)” is odds ratio and 95% confidence interval for an unusual IFN-γ measurement in QFT-G compared to referent, QFT-GIT.

e “**HNC**” is High Nil Concentration (i.e. [Nil] over 0.7 IU/mL).

f “**LMR**” is Low Mitogen Response (i.e. Mitogen Response under 0.5 IU/mL).

g “**VLMR**” is Very Low Mitogen Response (i.e. Mitogen Response ≤−0.5 IU/mL).

h “**VLAR**” is Very Low Antigen Response (i.e. response to a Mtb antigen ≤−0.35 IU/mL and ≤−0.5 times [Nil]).

**Table 3:** Subjects in Each Group with Unusual IFN-γ Measurements.

	Group	≥1 HNC^a^	≥1 LMR^b^	≥1 VLMR^c^	≥1 VLAR^d^
	Total	n (%)	n (%)	n (%)	n (%)
Employee Screening	864	35 (4.1)	12 (1.4)	0 (0)	12 (1.4)
Contacts	683	103 (15.1)	50 (7.3)	11 (1.6)	23 (3.4)
TB Suspect	181	24(13.3)	23 (12.7)	10 (5.5)	4 (2.2)
*p* value		<0.001	<0.001	<0.001	0.03

a “**HNC**” is High Nil Concentration (i.e. [Nil] over 0.7 IU/mL).

b “**LMR**” is Low Mitogen Response (i.e. Mitogen Response under 0.5 IU/mL).

c “**VLMR**” is Very Low Mitogen Response (i.e. Mitogen Response ≤−0.5 IU/mL).

d “**VLAR**” is Very Low Antigen Response (i.e. response to a Mtb antigen ≤−0.35 IU/mL and ≤−0.5 IU/mL).

### Frequency of Unusual IFN-γ Measurements Recurring with the Same Test Format

**Figure 2:**
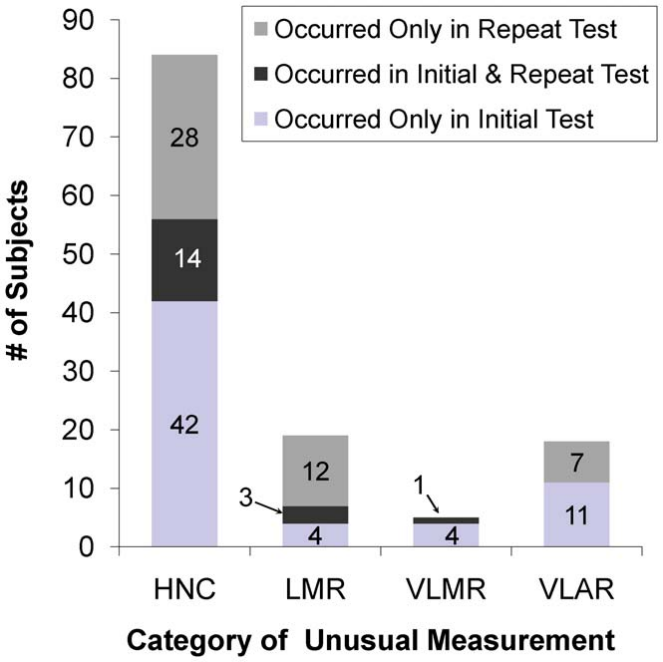
Number of subjects with unusual IFN-γ measurements when QuantiFERON-TB Gold tests were repeated. The number of subjects with unusual IFN-γ measurements categorized as High Nil Concentration (**HNC**), Low Mitogen Response (**LMR**), Very Low Mitogen Response (**VLMR**), and Very Low Antigen Response (**VLAR**) that occurred only with the initial test (light gray bars), with the repeat test (dark gray bars), or in both the initial and repeat test (medium gray bars) among 1,044 Subjects with Initial and Repeat QuantiFERON-TB Gold Test Results.

Of 1,045 subjects who had initial and repeat QFT-G, 56 subjects had **HNC** on initial QFT-G and 14 (25.0%) of these had a recurrent **HNC** (see Figure 2); 28 others had **HNC** only with repeat QFT-G**.** Of 7 subjects who had **LMR** on initial QFT-G, 3 (42.9%) had a recurrent **LMR**; 12 others had **LMR** only with repeat QFT-G. Five subjects had a **VLMR** on initial QFT-G and only 1 (20.0%) had a recurrent **VLMR**. While 11 subjects had a **VLAR** on initial QFT-G, none recurred (although 7 others had **VLAR** on repeat QFT-G and not on initial QFT-G).

**Figure 3:**
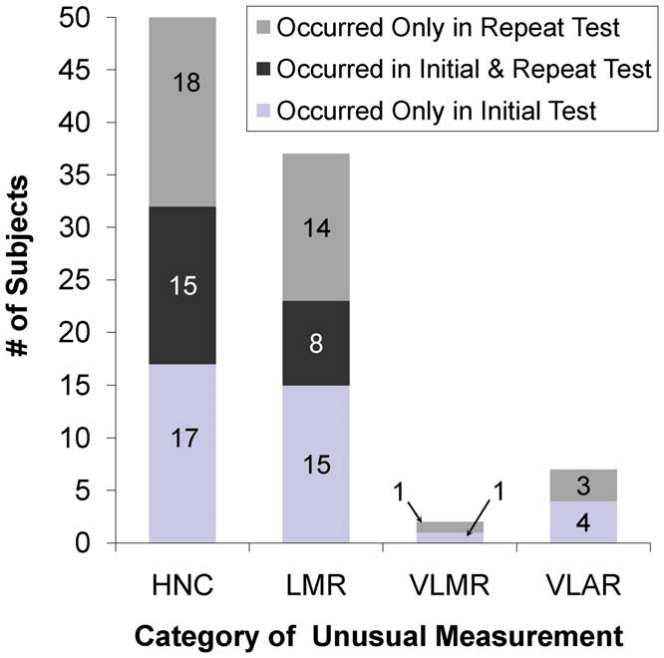
Number of subjects with unusual IFN-γ measurements when QuantiFERON-TB Gold In-Tube tests were repeated. The number of subjects with unusual IFN-γ measurements categorized as High Nil Concentration (**HNC**), Low Mitogen Response (**LMR**), Very Low Mitogen Response (**VLMR**), and Very Low Antigen Response (**VLAR**) that occurred only with the initial test (light gray bars), with the repeat test (dark gray bars), or in both the initial and repeat test (medium gray bars) among 915 subjects with initial and repeat QuantiFERON-TB Gold In-Tube test results.

Of 915 subjects who had initial and repeat QFT-GIT, 32 subjects had **HNC** on initial QFT-GIT, 15 (46.9%) had **HNC** recur, and 18 others had **HNC** occur only with repeat QFT-GIT (see Figure 3). Of 23 subjects who had **LMR** on initial QFT-GIT, 8 (34.8%) had a recurrent **LMR** and 14 had **LMR** occur only with repeat QFT-GIT. One person had a **VLMR** on the initial QFT-GIT test without recurrence; 4 subjects had a **VLAR** on initial QFT-GIT and none recurred.

### Frequency of Unusual IFN-γ Measurements with Simultaneously Performed IGRAs

**Figure 4:**
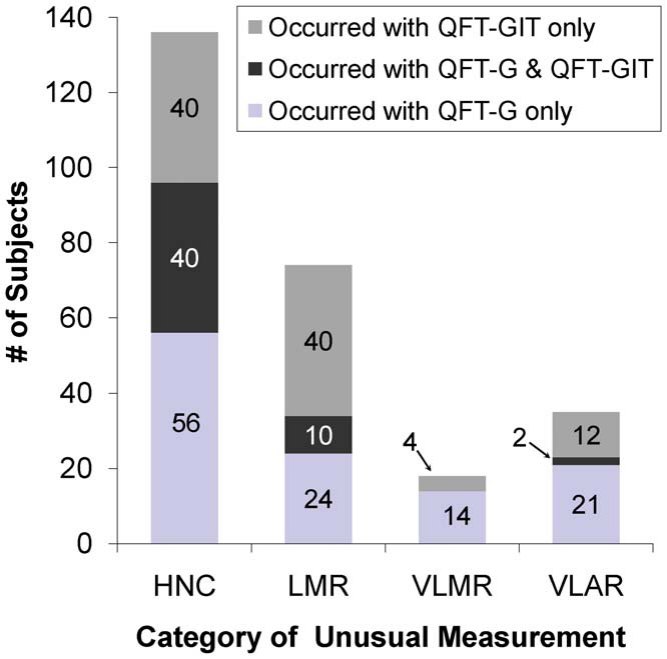
Number of subjects with unusual IFN-γ measurements when QuantiFERON-TB Gold and QuantiFERON-TB Gold In-Tube tests were run simultaneously. The number of subjects with unusual IFN-γ measurements categorized as High Nil Concentration (**HNC**), Low Mitogen Response (**LMR**), Very Low Mitogen Response (**VLMR**), and Very Low Antigen Response (**VLAR**) that occurred only with the QuantiFERON-TB Gold test (light gray bars), with the QuantiFERON-TB Gold In-Tube test (dark gray bars), or in both the QuantiFERON-TB Gold and QuantiFERON-TB Gold In-Tube test (medium gray bars) among 1,617 subjects with simultaneously performed QuantiFERON-TB Gold and QuantiFERON-TB Gold In-Tube Test.

Among 1,617 subjects who had simultaneously performed QFT-G and QFT-GIT, 56 subjects had **HNC** on QFT-G only, 40 had **HNC** on QFT-GIT only, and 40 (29.4%) of the 136 subjects with HNC on QFT-G or QFT-GIT had HNC on both tests (see Figure 4)**.** **LMR** was observed in both QFT-G and QFT-GIT for 10 (13.5%) of 74 subjects with **LMR** on simultaneously performed QFT-G or QFT-GIT. None of the subjects had **VLMR** with both QFT-G and QFT-GIT when preformed simultaneously. **VLAR** was observed in both QFT-G and QFT-GIT for 2 (5.7%) of 35 subjects with **VLAR** on simultaneously performed QFT-G or QFT-GIT.

### Frequency of Positive Results with Unusual IFN-γ Measurements

**HNC** occurred in 58 positive tests for 47 subjects, 24 (51.6%) of whom had negative or indeterminate results by all other IGRAs examined. **LMR** occurred in 21 positive tests for 21 subjects, 13 (61.9%) of whom had negative or indeterminate results by all other tests examined. One QFT-G with **VLMR** was interpreted as positive (due to an ESAT-6 response of 17.9 IU/mL) while the TB response measured by QFT-GIT performed at the same time was 0.01 IU/ml. No QFT-GIT with **VLMR** was interpreted as positive. Three QFT-Gs with **VLAR** were interpreted as positive, and in each case at least one other IGRA without unusual IFN-γ measurement was also interpreted as positive.

## Discussion

We found unusual IFN-γ measurements in 6.2% of QFT-G and 6.4% of QFT-GIT. Technical errors are a potential source for many of the unusual IFN-γ measurements we observed. Despite the potential advantages of using whole blood IGRAs to detect *Mtb* infection, these tests are more complex than the TST. While TST requires five measurements to complete one test (i.e. measurement of the volume of PPD to inject, the depth of injection, the time injected, the time delay until measuring induration, and measurement of induration size), QFT-G requires at least 136 measurements to complete one test (listed as supporting information in Table S1). The number of measurements required to complete one QFT-GIT is reduced to 126 (listed as supporting information in Table S2) by combining test antigens and including them in the tubes used to collect blood. With so many measurements and associated manipulations, it is easy to understand how technical errors could occur.

One scenario that could lead to **VLMR** for QFT-G or QFT-GIT is transposition of nil and mitogen IFN-γ measurements. Additional evidence of this possibility comes from examination of the Mitogen Response values. Of the 22 IGRAs with **VLMR**, 11 (50%) had a Mitogen Response <−10 IU/mL (data not shown). Similarly, **VLAR** could be due to transposition of measurements for nil and TB antigens. By eliminating the need to add antigens for QFT-GIT, the opportunity for transpositions is reduced, and we observed significantly less **VLMR** and somewhat less **VLAR** for QFT-GIT as compared to QFT-G. Additional evidence that some **VLMR** and **VLAR** are the result of technical errors is the rarity of their recurrence with subsequent testing. **VLMR** recurred in only one person and **VLAR** did not recur. Also, for most subjects with **VLMR** or **VLAR**, such unusual IFN-γ measurements were not seen on a simultaneously performed IGRA.

While **LMR** has been associated with immune suppression [10], [17], technical errors may produce indistinguishable results. If due to immune suppression, **LMR** would be expected on simultaneously performed tests, but only 13.5% of subjects who had **LMR** and a simultaneously performed IGRA had it on a the simultaneously performed IGRA. Additionally, **LMR** occurred in more than one test for only 21.2% of the subjects who had **LMR**.

The same transpositions that cause **LMR** and **VLMR** could account for a portion of the unusual IFN-γ measurements classified as **HNC**. However, in most situations, **HNC** occurred where transpositions were not suspected (i.e. without **LMR**, **VLMR** or **VLAR**). Other technical factors may be involved as is suggested by our observations that **HNC** occurred less often with QFT-GIT as compared to QFT-G, that **HNC** was rarely seen on two simultaneously performed IGRAs for the same person, and that **HNC** recurrence was uncommon. These findings suggest that tests with such unusual IFN-γ measurements should be repeated.

Sensitization to mouse antigens can generate heterophile antibodies in some people. These antibodies can bind to the capture and detection antibodies used in IGRAs and generate results consistent with **HNC**. Elevated levels of IFN-γ are seen in various infectious diseases (e.g., tuberculosis, acquired immunodeficiency syndrome, parasite diseases), autoimmune diseases (e.g., rheumatoid arthritis, thyroiditis, systemic lupus), and in allograft rejection. **HNC** would be expected to occur more frequently in patients with these conditions. Day-to-day variation in disease activity or changes in the amount of heterophile antibody present may explain some difference in **HNC** observed with initial and repeat testing. However, day-to-day variation would not explain difference in **HNC** observed when two IGRAs are performed simultaneously.

Technical errors appear to contribute to IGRA variability. However, data related to IGRA variability are scarce [18], [19]. This is in part because of the complexity of these tests. For example, reproducible QFT-GIT results require accurate measurement of [IFN-γ] in 3 samples in the correct order, and the use of multiple criteria for test interpretation. While testing samples by ELISA are traditionally performed in duplicate or triplicate, the manufacturer of QFT-G and QFT-GIT recommends testing once. Our review of results of multiple tests from the same person performed simultaneously and serially provided an opportunity to recognize unusual IFN-γ measurements, most of which were aberrant and seen only in one of multiple tests examined. The observation that most unusual IFN-γ are aberrant supports the recommendation to repeat testing using a fresh sample when unusual IFN-γ are encountered [2]. Additional studies comparing IGRA results performed multiple times on the same sample, performed on multiple samples collected at the same time, and performed on multiple samples collected at different times are needed to more fully assess IGRA reproducibility. Recognition of unusual IFN-γ measurements and the potential for technical errors will facilitate assessment of IGRA reproducibility.

QFT-G and QFT-GIT are occasionally interpreted as positive despite unusual IFN-γ measurements. In our study 21.4% of positive results were associated with unusual IFN-γ measurements. The observation that 51.1% of subjects with positive results associated with **HNC** and 61.9% of positive results associated with **LMR** had negative or indeterminate results by all other tests examined raises doubt as to their validity. It would seem that tests with unusual IFN-γ measurements should be interpreted as indeterminate regardless of the measured response to TB antigens.

Criteria for interpreting tests with high [Nil] have evolved. Initial criteria did not include an indeterminate category for tests with **HNC** [6], [9]. With FDA approval of QFT-G, indeterminate criteria were included for most tests with [Nil] over 0.7 IU/mL [4]. With FDA approval of QFT-GIT, indeterminate criteria were included for tests with [Nil] over 8.0 IU/mL [5]. Our observations suggest that raising the cutoff for identifying high [Nil] may result in an increased number of inaccurate interpretations.

While **HNC**, **VLMR**, and **VLAR** were more common with QFT-G than QFT-GIT, **LMR** was more common with QFT-GIT. One difference that may account for these observations is how blood is mixed with antigens. Including the antigens in the blood collection tubes reduces the complexity of QFT-GIT and reduces the opportunity for technical errors, but necessitates shaking the blood vigorously to dissolve the antigens and mix them with the blood. **LMR** may follow inadequate shaking due to incomplete integration of the mitogen with the blood, and excessive shaking may result in lysis of lymphocytes and reduced production of IFN-γ. Similar problems with tubes containing *Mtb* antigens could occur, but would be difficult to detect.

A limitation of this study is our inability to confirm the presence or absence of *Mtb* infection. We addressed this lack of an adequate diagnostic standard by comparing results of multiple tests from the same person performed simultaneously and serially.

In conclusion, unusual IFN-γ measurements such as **HNC**, **LMR**, **VLMR**, and **VLAR** were encountered in a small number of QFT-G and QFT-GIT, and in most cases, such measurements were not seen on simultaneously or subsequently performed tests. To avoid erroneous diagnosis of *Mtb* infection, QFT-G and QFT-GIT with unusual IFN-γ measurements should be repeated with another blood sample and interpreted with caution if they recur.

## Supporting Information

Table S1 **Measurements required for one QuantiFERON-TB Gold test.** The QuantiFERON-TB Gold test (QFT-G) requires at least 136 measurements to complete one test.
(DOC)

Table S2 **Measurements required for one QuantiFERON-TB Gold In-Tube Test.** The QuantiFERON-TB Gold In-Tube test (QFT-GIT) requires at least 126 measurements to complete one test.
(DOC)
