# Supplementary material for: Unusual Interferon Gamma Measurements with QuantiFERON-TB Gold and QuantiFERON-TB Gold In-Tube Tests
Source: PLoS One. 2011 Jun 8;6(6):e20061. doi: 10.1371/journal.pone.0020061 (PMC3110578; doi:10.1371/journal.pone.0020061)
Supplement: Table S2 — Measurements required for one QuantiFERON-TB Gold In-Tube Test. The QuantiFERON-TB Gold In-Tube test (QFT-GIT) requires at least 126 measurements to complete one test. (DOC) [file pone.0020061.s002.doc]

**Table S2: Measurements required for one** QuantiFERON-TB Gold In-Tube Test

| Number of Measurements | Description of Measurement |
| --- | --- |
| 1 | Time blood was collected |
| 3 | Collect 0.8 to 1 mL of blood into 3 tubes |
| 2 | Time of onset and end of incubation |
| 1 | Incubator temperature (37.0 +/- 1.0ºC) |
| 1 | 300 µL of H2O to reconstitute Conjugate |
| 1 | Designated volume of H2O to reconstitute IFN-γ standard |
| 7 | 300 µL of Green Diluent for 7 standards |
| 1 | 300 µL of stock standard for 1st standard |
| 6 | 300 µL of standards transferred for serially dilution |
| 1 | Designated volume of Green Diluent for conjugate solution |
| 1 | Designated volume of Conjugate concentrate for conjugate solution |
| 19 | 50 µL of conjugate solution to control wells (16) & samples (3) |
| 2 | Time of onset and end of 2 hour ELISA incubation |
| 1 | Designated volume of Wash Concentrate for Wash solution |
| 1 | Designated volume of H2O for Wash solution |
| 19 | 100 µL of substrate to each control and sample well |
| 2 | Time of onset and end of 30 min incubation with substrate |
| 19 | 50 µL of Stop solution to each control and sample well |
| 38 | Optical density of control and sample well at 450 & 630 nm |
| **126** | **TOTAL Measurements** |
